# Anti-breast cancer action of carbonic anhydrase IX inhibitor 4-[4-(4-Benzo[1,3]dioxol-5-ylmethyl-piperazin-1-yl)-benzylidene-hydrazinocarbonyl]-benzenesulfonamide (BSM-0004): *in vitro* and *in vivo* studies

**DOI:** 10.1080/14756366.2021.1909580

**Published:** 2021-05-05

**Authors:** Chandra Bhushan Mishra, Raj Kumar Mongre, Amresh Prakash, Raok Jeon, Claudiu T. Supuran, Myeong-Sok Lee

**Affiliations:** a College of Pharmacy, Sookmyung Women’s University, Seoul, Republic of Korea; b Department of Biosystem, Molecular Cancer Biology Laboratory, Cellular Heterogeneity Research Center, Sookmyung Women’s University, Seoul, Republic of Korea; c Amity Institute of Integrative Sciences and Health, Amity University, Gurgaon, India; d Dipartimento Neurofarba, Sezione di Scienze Farmaceutiche e Nutraceutiche, Universitàdegli Studi di Firenze, Florence, Italy

**Keywords:** Human carbonic anhydrase IX, inhibition constant, breast cancer, cancer therapy, cytotoxicity

## Abstract

Anti-breast cancer action of novel human carbonic anhydrase IX (hCA IX) inhibitor BSM-0004 has been investigated using *in vitro* and *in vivo* models of breast cancer. BSM-0004 was found to be a potent and selective hCA IX inhibitor with a Ki value of 96 nM. *In vitro* anticancer effect of BSM-0004 was analysed against MCF 7 and MDA-MA-231 cells, BSM-0004 exerted an effective cytotoxic effect under normoxic and hypoxic conditions, inducing apoptosis in MCF 7 cells. Additionally, this compound significantly regulates the expression of crucial biomarkers associated with apoptosis. The investigation was extended to confirm the efficacy of this hCA IX inhibitor against *in vivo* model of breast cancer. The results specified that the treatment of BSM-0004 displayed an effective *in vivo* anticancer effect, reducing tumour growth in a xenograft cancer model. Hence, our investigation delivers an effective anti-breast cancer agent that engenders the anticancer effect by inhibiting hCA IX.

## 1. Introduction

Breast cancer is the most prevalent type of cancer that affects millions of people globally and is correlated with the main cause of mortality in women aged between 45 and 55 years^1^^,^^2^. It is a type of tissue cancer that mainly arises from the inner layer of lobules and ducts. Breast cancer is a multistage disease and it reaches the metastasis stage in advanced cases^3^^,^^4^. The main risk factors of this disease include age, high hormone level, bad lifestyle, iodine deficiency, and economic status^5^^,^^6^. The therapy of breast cancer mainly relies on tissue removal, hormone therapy, and chemotherapy^7^^,^^8^. In the past decades, enough progress has been made for successful therapy of breast cancer. Unfortunately, several cases of breast cancer are still incurable, especially advanced-stage breast cancer^9^^,^^10^.

The carbonic anhydrases (CAs) belong to the zinc metalloenzymes category and catalyse the reversible reaction of carbon dioxide and water to form bicarbonate^11–14^. CAs actively partake in crucial physiological processes such as pH regulation, water, and electrolyte balance as well as CO_2_ and HCO_3_^–^ transport^15–18^. Among 15 isoforms of CAs, CA IX is extensively studied as an effective target for the therapy of hypoxic cancers^19–23^. Human carbonic anhydrase IX (hCA IX) has 459 amino acid residues, consisting of an extracellular part, an N-terminal signal peptide, a transmembrane region, and an intracellular part. The extracellular part contains a catalytic domain, situated nearby the plasma membrane (displaying high sequence homology with other hCA isoforms) and a proteoglycan (PG)-like domain that is considered as a unique property of CA IX, it is found to be absent in other isoforms^19^^,^^24–26^. CA IX gene is associated with hypoxic tumour cells and codes a transmembrane glycoprotein consisting of an extracellular catalytic domain as well as N-terminal PG domain, regulated by hypoxia-inducible factor-1 (HIF-1)^27^^,^^28^. CA IX is widely expressed in various types of solid tumours and promotes the progression of tumours as well as their metastasis by generating an acidic environment in tumour cells^29–31^. Under normal physiological conditions, very limited expression of CA IX was observed and found mainly in intestine and gallbladder epithelia^32^^,^^33^. However, overexpression of hCA IX has been observed in a variety of malignant cancers such as breast, head and neck, renal, colon, lung, oesophagus, ovary, vulva, and brain tumors^20^^,^^34–37^. Thus, CAs actively participate in maintaining physiological pH in cancer cells and acidic pH in the extracellular part that are considered as a favourable environment for the proliferation of cancer cells and their metastasis^38^^,^^39^. It has been found that the catalytic domain of hCA IX that is present on the extracellular part of the cell is actively involved in this process, maintaining the concentration of proton and bicarbonate^40^^,^^41^. It has been studied that CA IX promotes acidic pH of tumour cells in a culture medium and its inhibition decreases survival of cancer cells under hypoxia condition^42^^,^^43^. The function of CA IX in cancer cell progression and migration is also correlated with the Rho/ROCK signalling pathway^44^. The investigations also directed that CAIX may influence cell adhesion pathways to promote cancer cell survival and proliferation^45^. Thus, hCA IX emerged as an effective druggable target to control cancer cell proliferation and metastasis^19^. In order to target hCA IX for the management of several cancers, numerous varieties of small molecules have been developed as effective hCA IX inhibitors^19^^,^^46^. Among all categories, sulphonamides emerged as a very effective class of hCA IX inhibitors^47^^,^^48^.

Over the last decades, several sulphonamides containing hCA IX inhibitors have been developed, with excellent *in vitro* and *in vivo* anticancer activity^19^. Interestingly, ureido-benzenesulfonamide hCA IX inhibitor SLC-0111 emerged as an effective anticancer agent, showing efficacy against various types of cancers and is currently in phase Ib/II clinical trials^49^. However, most other hCA IX inhibitors with effective anti-cancer activity are under the preclinical evaluation stage and very few hCA IX inhibitors are running in the clinical trial study which may hamper the progress of hCA IX targeted anti-cancer drug development^19^^,^^50^. Therefore, effective hCA IX inhibitors with promising anticancer activity are needed to explore this target for the management of other types of cancer. Recently, we have developed benzene sulphonamide based hCAs inhibitors using substituted piperazine tails with benzylidenehydrazine carbonyl spacer. Among them, 4-[4-(4-Benzo[1,3]dioxol-5-ylmethyl-piperazin-1-yl)-benzylidene-hydrazinocarbonyl]-benzenesulfonamide (BSM-0004) appeared to be an effective and selective inhibitor for hCA IX^13^. In the present work, the anti-breast cancer activity of BSM-0004 has been carried out against MCF7 and MDA-MA-23 cells under normoxic as well as hypoxic conditions. In order to explore the molecular mechanism of this novel hCA IX inhibitor, several *in vitro* experiments have been performed. Additionally, *in vivo* anti-breast cancer activity of BSM-0004 has been assessed, using xenograft nude mice model.

## 2. Materials and methods

### 2.1. Cell line and culture conditions

MCF-7 cells were procured from ATCC (Manassas, VA) and maintained in complete DMEM media. Approximately, 10% foetal bovine serum (FBS; Hyclone, Logan, UT) and 1% antimycotic and antibiotic (Cat. No. 15240-06; Gibco, Gaithersburg, MD) were added to prepare complete DMEM medium. The cells were grown in an undeviating system of 95% air, and 5% CO_2_ at the 37 °C.

### 2.2. Cytotoxicity and proliferation assay

Cytotoxic effect of BSM-0004 against breast cancer MCF7 and MDA-MA-231 cells was investigated using 3-(4,5-dimethylthiazol-2-yl)-2,5-diphenyltetrazolium bromide (MTT) reduction assays. Totally, 1.0 × 10^5^ cells were seeded in 96-well plates for overnight incubation with complete DMEM medium and subjected to the experimental treatments as indicated. Twenty-four hours post-drug treatments, the viable cells were stained by adding 25 μL of freshly prepared MTT solution (5 mg/mL in PBS) in to individual wells (containing 100 μL cell culture media) and incubated at 37 °C for 4 h. After incubating for 4 h at 37 °C, the media were removed and 150 μL DMSO was added to dissolve the formazan. Following 30 min incubation at room temperature in a humidified chamber, colourimetric measurements were done using a multi-well microplate reader (LEDETECT96 LED based 8 channel Microplate Absorbance Reader). Efficacy of BSM-0004 and morphological analysis were also observed by optical microscope and captured cells morphology images in a different dose^51–54^.

### 2.3. Colony formation assay

About 500 cells resuspended in DMEM complete medium containing 10% FBS were plated on each well of six-well plates. The plates were incubated with or without 200 µM CoCl_2_ and treated with 10 and 20 µM BSM-0004 and propagated under the steady state conditioned with 37 °C in a 5% CO_2_ incubator for nine days. The total number of colonies was stained with crystal violet dye solution for 2 h. The stained colonies greater than 20 μm in diameter were counted using the ImageJ software (Bethesda, MD).

### 2.4. Early and late apoptosis analysis

MCF7 cells were thawed and cultured overnight, next day the cells were collected using trypsin–EDTA (0.25% Thermo Fisher Scientific Co., Seoul, Korea), then mixed with 3 mL of DMEM to seed in 60 mm cell-culturing round petri-dishes for 24 h. Next day, cells were treated with 10 and 20 µM of BSM-0004 with or without 200 µM of CoCl_2_. Followed by incubation with 24 h, total 1 × 10^6^ cells from each sample were collected and washed with cold PBS. In order to analyse total apoptotic cells in each sample, 5 μL of Annexin V-FITC and propidium iodide (PI) (Kit, AntGene, ant003, Wuhan, China) were added and mixed properly then incubated at RT for 30 min. Before analysis, 500 μL 1× binding buffer was added then cells were quantified for apoptosis using BD FACSCanto^TM^ II Cell Analyser (Becton-Dickinson, San Jose, CA).

### 2.5. Flow cytometric analysis for cell cycle arrest

The sub-population of cells in various cell cycle phase was investigated by staining with PI. The cell checkpoints were observed using BD FACSCanto^TM^ system according to reported previously^51–54^. Following to the treatment with 10 and 20 µM (with or without 200 µM CoCl_2_) concentrations of BSM-0004 for 24 h, approximately ∼1 × 10^6^ cells MCF7 cells were harvested and fixed with 70% ethanol at room temperature by gently vortexed and kept at 4 °C for overnight. The fixed cells were rinsed two times with PBS. The PI-staining has been carried out to detect the percentages of cells in different checkpoints as G1, S, and G2/M phases of the cell cycle and the signals were detected using FL2-channel.

### 2.6. Protein extraction and western blot analysis

For the protein estimation, MCF7 cells were cultured in DMEM complete medium with 10 and 20 µM concentration of BSM-0004 and DMSO (control group), respectively. Next day, cells were harvested using pellet downed by centrifugation at 1000 rpm (Hanil Scientific, Inc., Gimpo, South Korea) for two minutes at 4 °C. Then, cells were rinsed with 1× PBS and followed the instruction as reported previously^51–54^. Briefly, cells pellet was spin-down and lysed in the RIPA lysis buffer with DTT, NaVO3, and PI, respectively. The Bradford method was used to quantify the total protein concentration, after that 5× protein sample buffer was properly added to the protein lysate then boiled at 95 °C for 10 min. The PVDF membranes were blotted with mouse anti-BAX (1:1000), and anti-CA-IX (1;500), rabbit polyclonal anti-XIAP (1;1000), anti-Bcl2 (1:500), anti-STAT-3 (1:1000), and anti-pSTAT-3 (1:500). The PVDF membranes were transferred in rotating shaker at 4 °C for immunoblotted against anti-mouse and anti-rabbit IgG (1:2000 dilution, Santa Cruz Bio Inc., Santa Cruz, CA). The immunoblot levels of proteins were observed using chemiluminescence system and relative band intensities were calculated using ImageJ software (Bethesda, MD).

### 2.7. Tumoricidal role of BSM-0004 in xenograft model of nude mice

The immunodeficient 5 weeks old female nude mice were procured from ORIENT BIO Inc. (Seongnam-si, South Korea). The nude mice were acclimatised in suitable condition and provided enough water and food routinely. The full experimental plan and methods were designed and approved by the Sookmyung Women’s University’s Board of Review Committee, and the Department of Animal Facility, Yongsan-gu, Seoul, Republic of Korea. The efficacy of BSM-0004 against MCF7 induced tumour burden in nude mice was carried out as described previously^51–54^. Briefly, totally 15 mice were sub-grouped in control (*n* = 6), treatment (*n* = 6), and normal (*n* = 3) mice group, respectively. To generate tumour, 3 × 10^6^ cells were injected (by needle syringe, 27 G/12.7 mm, Ultra-Thin Plus™, Seoul, South Korea) onto left and right flank of nude mice, then the treatment group mice were administered with 15 mg/kg BSM-0004 and control group mice were treated with DMSO. After 4th post days of inoculation, tumours were visible and started measuring the tumour volume followed with volume = 1/2 × length × width^2^. Lastly, after finishing the experiment the mice were sacrificed followed by Sookmyung Women’s University’s standard protocol.

### 2.8. *In vivo* histopathological analysis of tumour samples

The efficacy of BSM-0004 and oncogenic expression of tumour marker Ki67 and hCA-IX were evaluated by immunohistochemistry of tumour samples as described in the previous study^51–54^. Briefly, tumour tissues were fixed with 4% paraformaldehyde then embedded with high grade of paraffin. The tissue-sections were cut in 5 µm thick using a microtome (Leica RM2255 Fully Automated Rotary Microtome, Wetzlar, Germany), then sliced tissues were fixed onto glass slides and deparaffinised with various percentage of xylene subjected to rehydrated with different concentrations of alcohol (C_2_H_5_OH). All slides were incubated with methanol containing 3% of hydrogen peroxide to be retrieved. After blocking with 5% horse serum, the slides were incubated with rabbit polyclonal antibodies for Ki67 (diluted, 1:400; Goat poly, Santa Cruz, Santa Cruz, CA) and anti-CA-IX (1:500 diluted, Mouse, abcam ab107257, Cambridge, UK). The immunohistopathological expression of Ki67 and hCA-IX was counter stained with biotinylated anti-rabbit and anti-mouse IgG (Vector Laboratories, Burlingame, CA). The diaminobenzidine (DAB) substrate was used to develop and precipitate the protein. Lastly, Mayer’s haematoxylin blue staining was performed to distinctly observe nuclear portion. The Ki67 and hCA-IX protein levels were detected using Leica microscope system (Wetzlar, Germany).

### 2.9. Statistical analysis

All data were analysed using the GraphPad Prism5 software (GraphPad Prism Software, La Jolla, CA). Data were obtained and mean ± SD used for significant analysis. The Mann–Whitney test was performed to analyse the tumour weight in BSM-0004 treated vs. control group. On the other hand, rest of experiments were analysed using the one-way analysis of variance (ANOVA) test was performed to elucidate statistical differences and *p*< .05 was opted as a statistically significant.

## 3. Results

### 3.1. BSM-0004 strongly inhibits hCA IX

**Figure 1.**
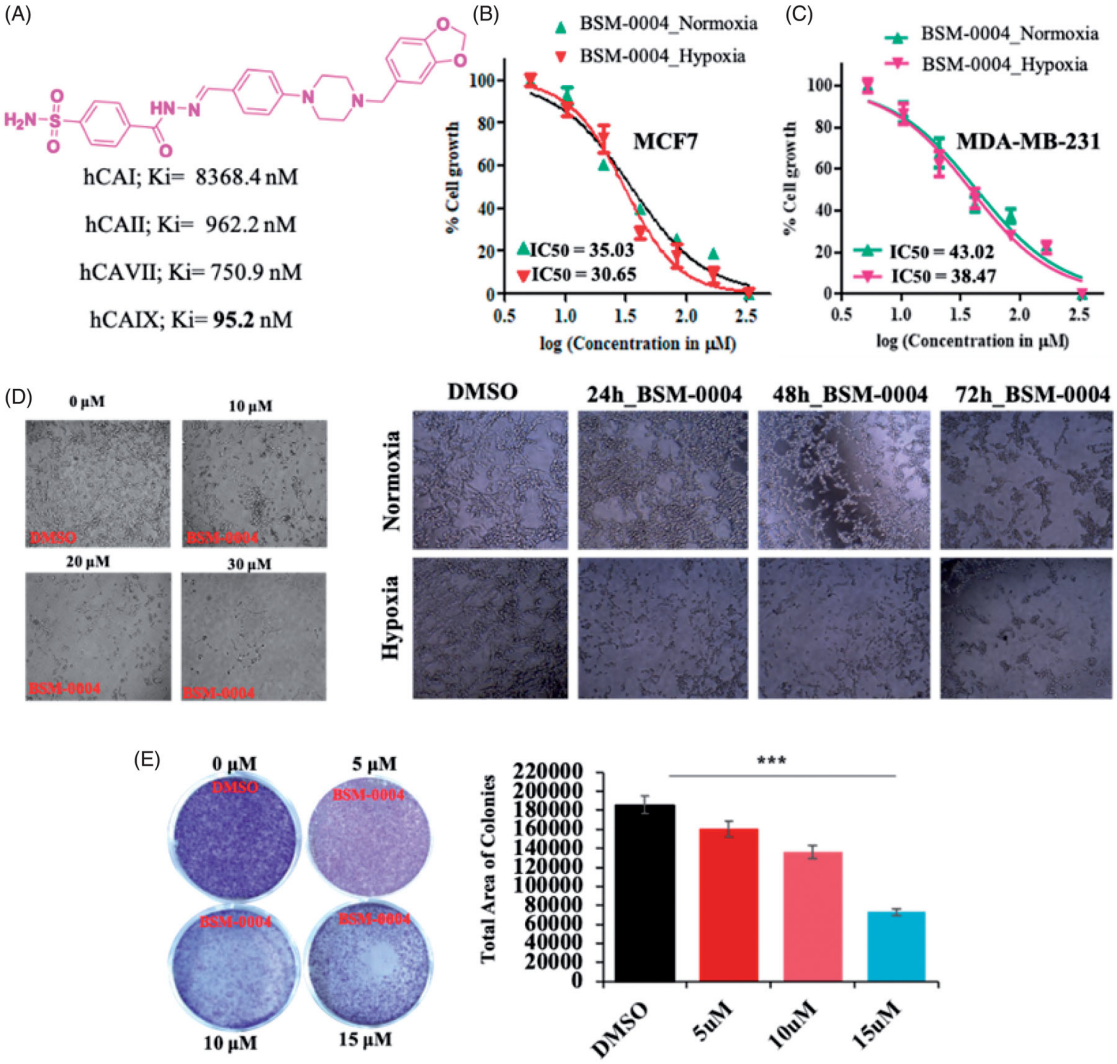
Tumoricidal role of BSM-0004 against MCF7 cells. (A) Inhibitory action of BSM-0004 against hCA I, II, IX, and XII isoforms. (B) BSM-0004 significantly inhibits the propagation of MCF7 cells. IC_50_ curves were fitted to the data. (C) BSM-0004 significantly impedes the propagation of MDA-MA-231cells in normoxic and hypoxic conditions. IC_50_ curves were plotted using obtained data. (D) Concentrations (10, 20 and 30 μM) and time dependent (24–72 h) morphological changes in MCF-7 cells were observed under the phase-contrast microscopy after treatment of BSM-0004. (E) The influence of BSM-0004 on the clonogenicity of MCF7 cells; the cells were seeded in six-well plates and treated with indicated concentrations of BSM-0004 for eight days, total number of colonies were calculated using ImageJ (Bethesda, MD).

*In vitro* CA inhibition study indicates that BSM-0004 effectively inhibits cancer-associated hCA IX in the nanomolar range. The result displayed that BSM-004 showed a Ki value of 95.1 nM against hCA IX. However, BSM-0004 displayed a Ki value of 8368.4, 962.2, and 750.9 nM against hCA I, hCA II, and hCA VII, respectively, that indicates selective inhibition towards hCA IX^13^. Hence, BSM-0004 emerged as a selective hCA IX inhibitor over other isoforms (Figure 1(A)).

### 3.2. Cytotoxic effect of BSM-0004 against MCF7 and MDA-MA-231 cells under normoxic and hypoxic conditions

*In vitro* cytotoxic effect of BSM-0004 was assessed against MCF7 and MDA-MA-231 cells under hypoxic and normoxic conditions. The result directed that BSM-0004 showed effective cytotoxicity against both cell lines under hypoxic as well as normoxic conditions (Figure 1). BSM-0004 showed IC_50_ values of 35.03 and 30.65 µM under normoxic and hypoxic conditions, respectively, against MCF7 cells as compared to standard drug doxorubicin which has shown an IC_50_ value of 21.90 µM^51^ in normoxic condition (Figure 1(B)). However, IC_50_ values of 43.02 and 38.47 µM were displayed by BSM-0004 against MDA-MB-231 cells in normoxic and hypoxic conditions, respectively (Figure 1(C)). Indeed, BSM-0004 showed a significantly effective cytotoxic effect against MCF-7 cells as compared to MDA-MA-231 cells in normoxic as well as hypoxic conditions. Additionally, a phase contrast microscopy study also authenticated the cytotoxicity efficacy of BSM-0004 against MCF-7 cells where significant cell death was visualised with the treatment of BSM-0004. Moreover, treatment of BSM-0004 also inhibited colony formation of MCF-7 cells in a concentration-dependent manner that further confirmed the anti-proliferative action of BSM-0004 against MCF7 cells (Figure 1(D,E)).

### 3.3. BSM-0004 arrests G2/M phase of cell cycle and persuades apoptosis

**Figure 2.**
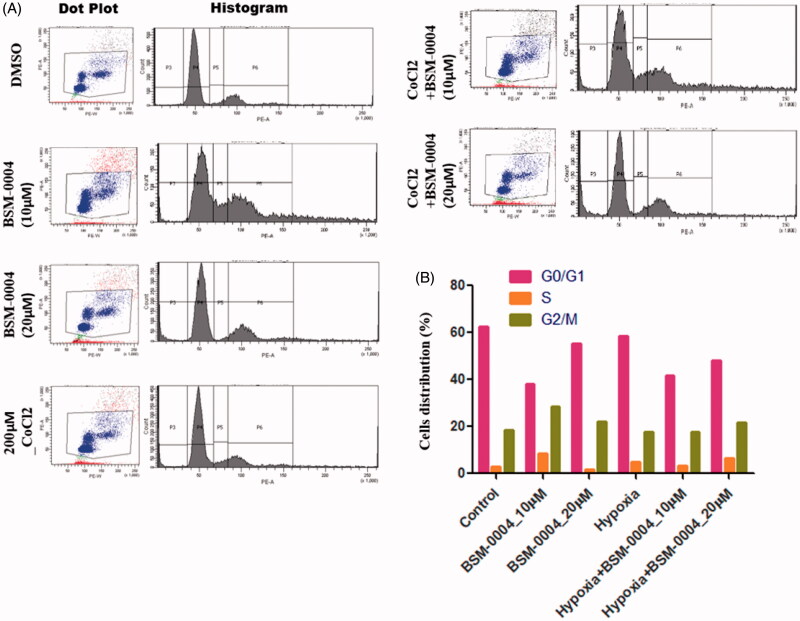
BSM-0004 mediated cell cycle arrest in MCF7 cells by FACS. (A) The cell cycle inhibition activity of BSM-0004; MCF7 cells were cultured under the exposure of vehicle (DMSO; control), 10 μM, and 20 μM BSM-0004 for 24 h under normoxia and hypoxia (CoCl_2_) conditions, cells arrest was determined according to flow cytometer instructions. In the histogram peak, the *x*-axis shows the total content of DNA by staining with propidium iodide, on the other hand, *y*-axis denotes the total number of MCF7 cells.

**Figure 3.**
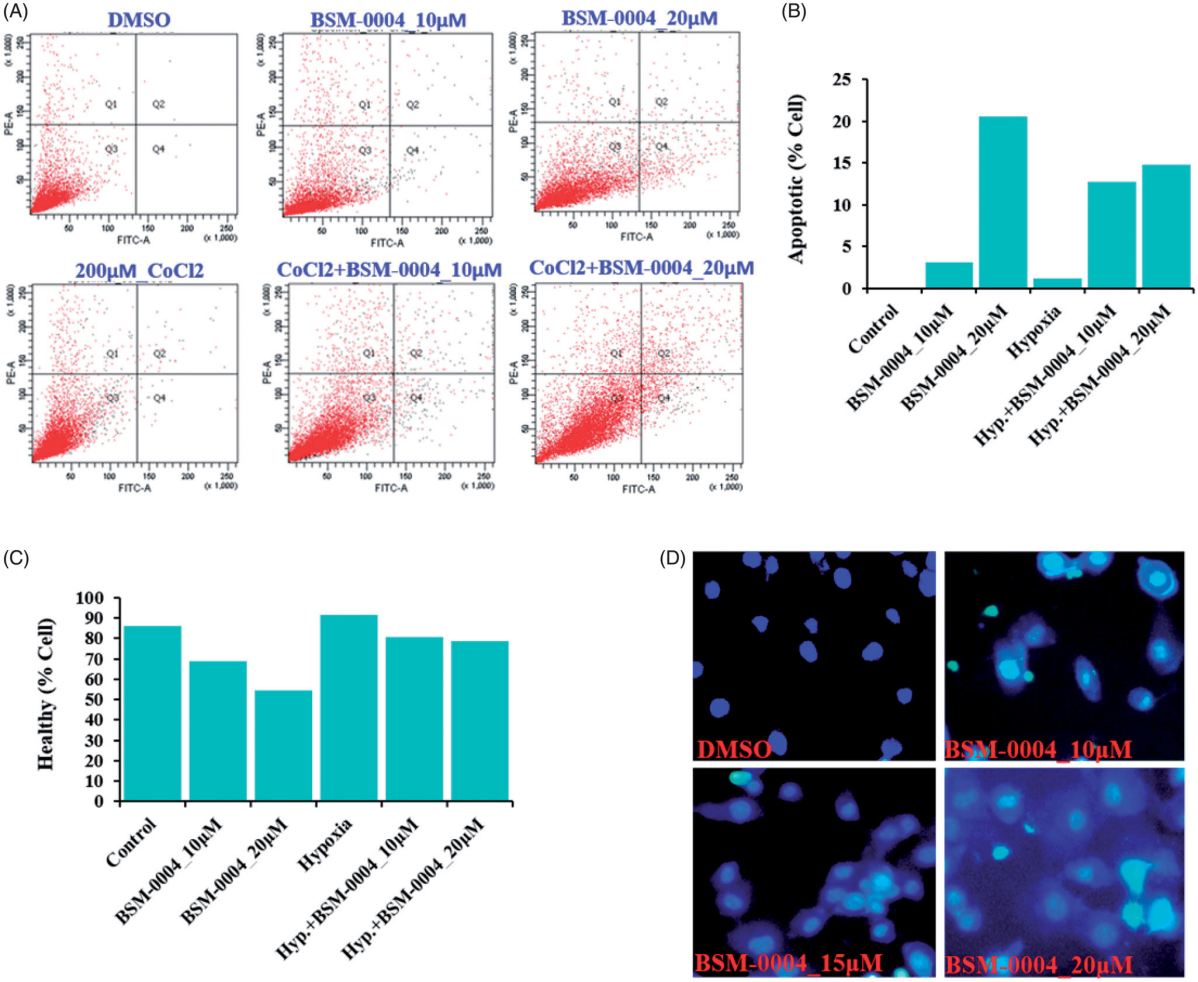
BSM-0004 induces pre/late apoptosis. (A–C) The apoptotic MCF-7 cells were measured by positive double staining of Annexin-V-FITC/PI. Apoptosis was tested by FACS analysis after the cells were treated with BSM-0004 (with or without CoCl_2_) for 24 h. (D) Nuclear fragmentation of apoptotic MCF7 cells was observed using DAPI staining. Cells were treated with BSM-0004 at concentrations of 10, 15, and 20 μM for 48 h, then cells were stained by DAPI and observed by fluorescence photomicrography (×200).

A flow-cytometric analysis was conducted to examine the cell cycle arresting potential of BSM-0004 at 10 and 20 µM concentrations under normoxia and hypoxia conditions. The result evoked that BSM-0004 efficiently arrested the G2/M phase of the cell cycle and significantly increased the population of MCF7 cells, concentration-dependently as compared to control. In normoxia condition, BSM-0004 displayed 28.6 and 22.2% cell population in G2/M phase at 10 and 20 µM concentration, respectively, whereas untreated cells showed 18.3% cells in this phase (Figure 2(A)). However, in hypoxia condition, 17.8 and 21.8% cell population were seen in the G2/M phase after treatment of BSM-004 at 10 and 20 µM concentrations, respectively, as compared to untreated cells that exhibited 17.6% cells (Figure 2(A)). Further, we also confirmed the apoptotic action of BSM-0004 in MCF7 cells by double staining method using PI and Annexin-V-FITC (Figure 3(A,B)). As displayed in Figure 3(A–C), BSM-0004 treatment at the concentration of 10 and 20 µM exerted 3.1 and 20.5% apoptosis, respectively, in MCF7 cells compared to control (showing 0.1%) under normoxic condition, while 12.7 and 14.8% apoptotic cells were observed at 10 and 20 µM concentrations in hypoxic condition as compared to control (showing 1.2%). In addition to this, the apoptotic effect of BSM-0004 was also confirmed by DAPI staining, where significant nuclear fragmentation was pictured upon treatment of BSM-0004 to MCF7 cells as compared to control (Figure 3(C)).

### 3.4. BSM-0004 impedes MCF-7 cells growth targeting CA IX

**Figure 4.**
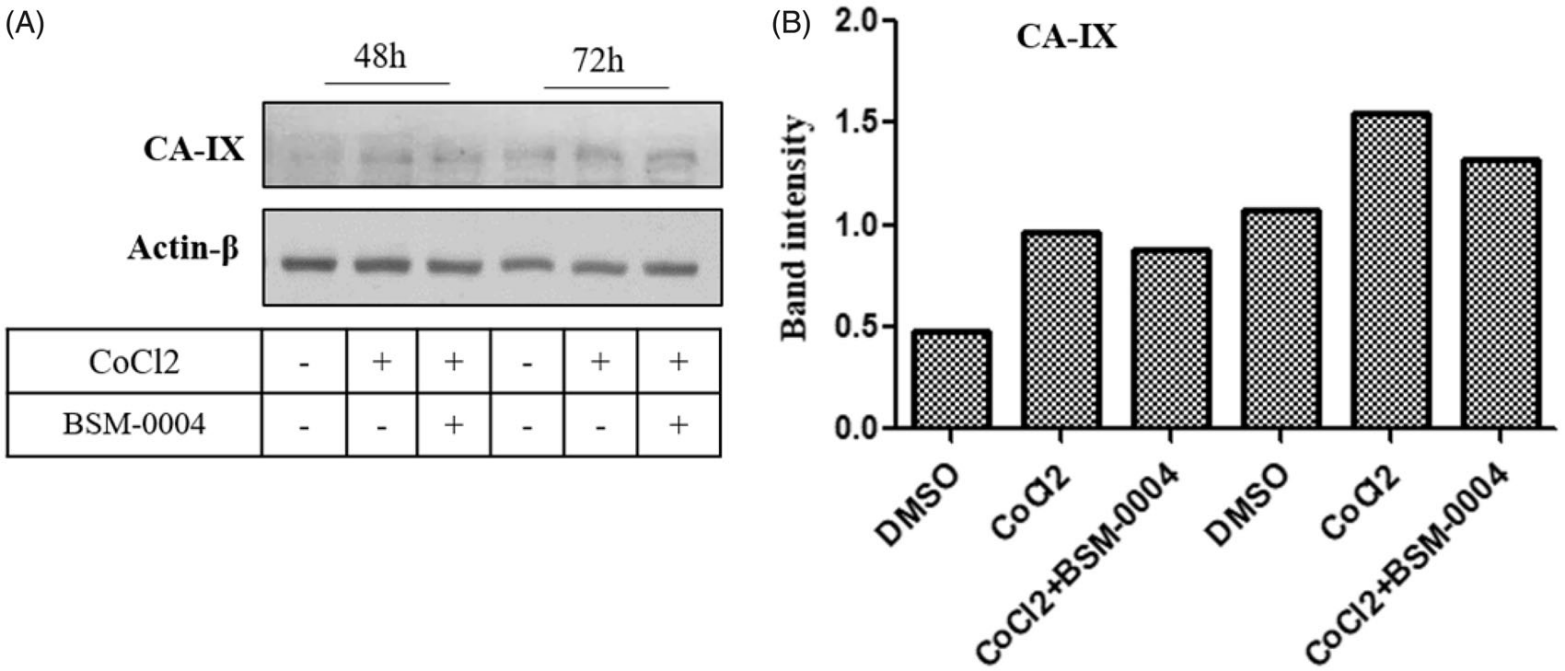
Activation and inhibition of hCA IX under the normoxic and hypoxic conditions. (A) Inhibition of the activated of hypoxia-induced hCA IX by BSM-0004 in MCF7 cells. (B) Band intensity was analysed using ImageJ (Bethesda, MD).

Our result indicated hypoxia significantly enhanced the expression of hCA IX in a time-dependent manner as compared to normoxic cells. Interestingly, treatment of BSM-0004 significantly reduced hypoxia-induced hCA IX expression in MCF7 cells time-dependently. However, a significant reduction in expression of hCA IX was observed in 72 h of BSM-0004 (Figure 4). Thus, this study indicates that BSM-0004 induced apoptosis by targeting hCA IX as observed in hCA inhibition study in which BSM-0004 effectively inhibited hCA IX, displaying nanomolar range Ki value.

### 3.5. Effect of BSM-0004 on apoptotic biomarkers

**Figure 5.**
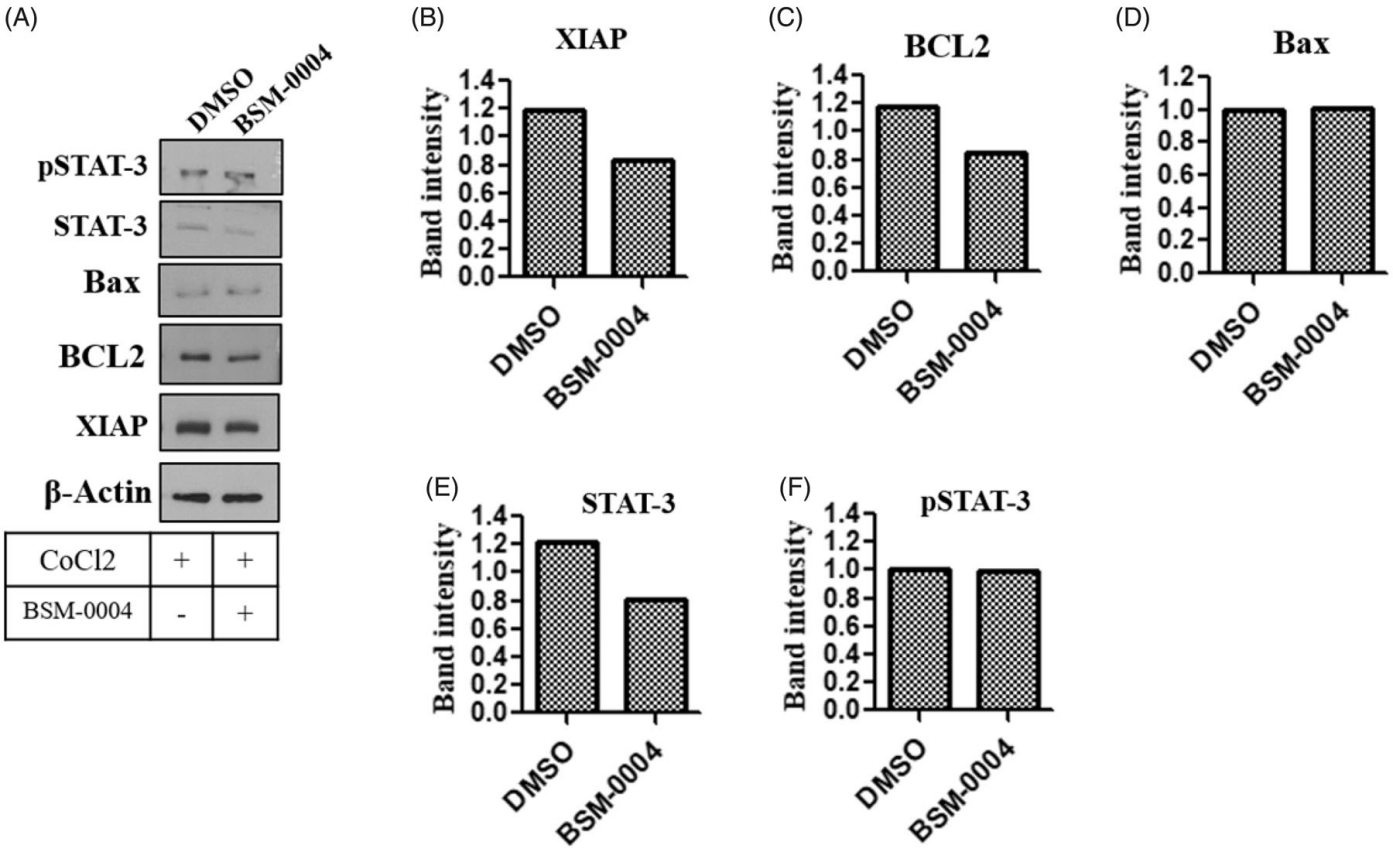
Novel inhibitor of hCA-IX BSM-0004 inhibits MCF7 cells growth by activation of apoptotic signalling cascade. (A) BSM-0004 inhibits cancer growth of MCF7 through apoptosis pathway. Immunoblot study was done to investigate the protein levels of apoptosis markers as XIAP, BCL-2, BAX, STAT-3, and pSTAT-3. B-F relative band intensity of XIAP, BCL-2, BAX, STAT-3, and pSTAT-3 were analysed using ImageJ (Bethesda, MD).

To confirm the effect of BSM-001 treatment on various apoptotic biomarkers such as Bcl2, XIAP, p-STAT-3, and STAT-3, a western blot study was performed. Our investigation showed that treatment of BSM-004 significantly decreases the expression of Bcl2 under the hypoxic condition as compared to untreated cells. Additionally, another anti-apoptotic biomarker XIAP was also found to be downregulated after treatment of BSM-0004 as compared to control. Moreover, treatment of BSM-0004 also down regulated transcriptional factor STAT-3 as compared to untreated cells. Hence, these results evidently indicate that BSM-0004 effectively regulates apoptotic biomarkers associated with apoptosis, which leads to induce cell death in MCF-7 cells (Figure 5(A–F)).

### 3.6. BSM-0004 obstructs tumour growth *in vitro* and *in vivo*

**Figure 6.**
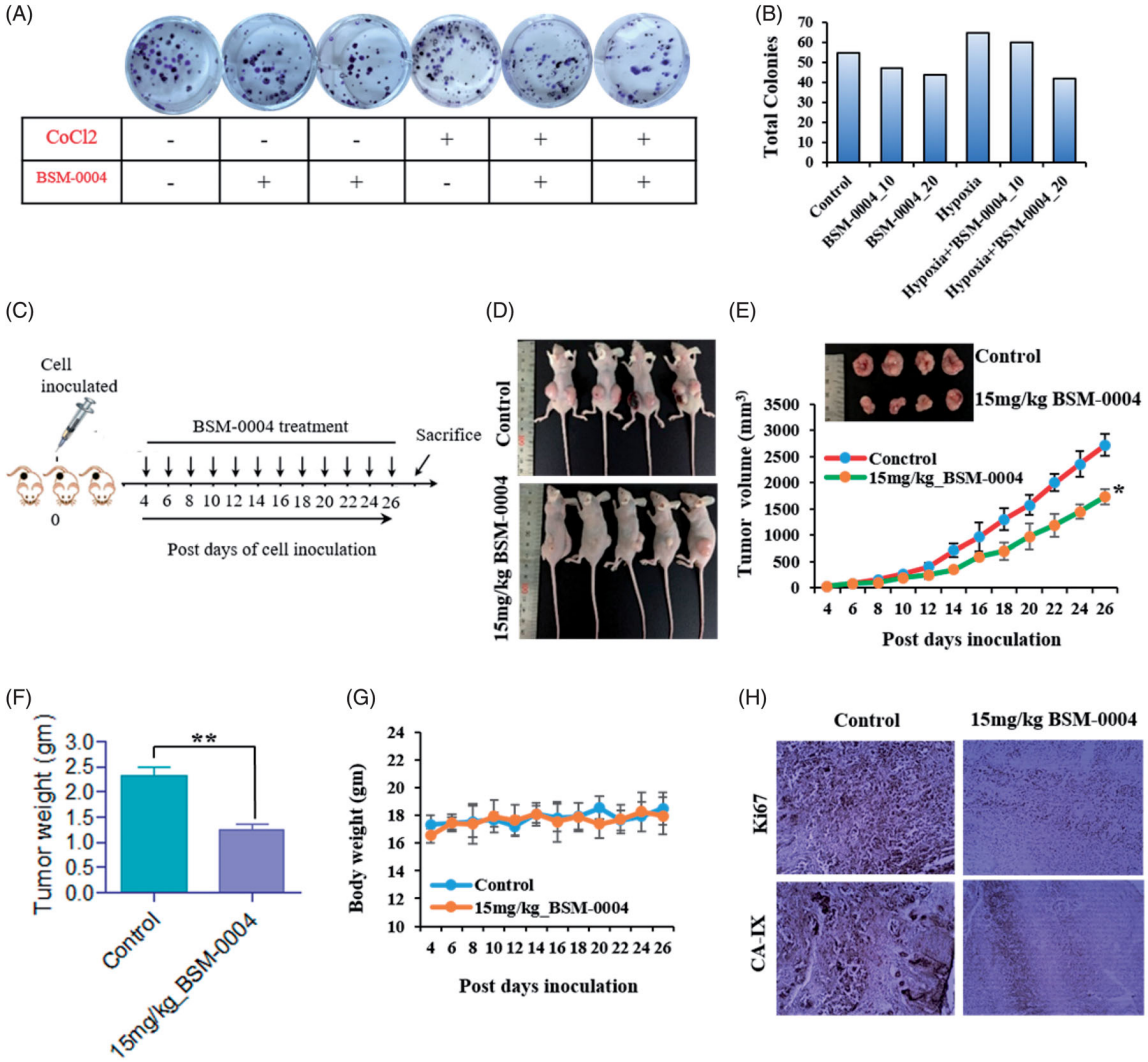
BSM-0004 inhibits tumour growth *in vitro* and xenograft nude mouse model. (A) Clonogenic activity of MCF7 cells was inhibited by long-term treatment of BSM-0004. (B) The total colonies were counted using ImageJ software (Bethesda, MD). (C) Diagrammatic presentation of establishment of xenograft and treatment with 15 mg/kg BSM-0004. (D) BSM-0004 inhibits MCF7 inoculated tumour growth in nude mice xenograft model. (E) Total 2.5 × 10^6^ MCF7 cells were inoculated in right/left flanks of BALB/c nude mice. After 4th days of injection, mice treated with 15 mg/kg of BSM-0004 every second day of the experiment. Tumour volume was measured every second day. (F) Each bar represents the mean ± standard error of mean (SEM) of the tumour weight of four groups. (G) The mice body weight (g) was observed in every second day of the experiment. (H) Immunohistochemistry (IHC) staining for hCA IX and Ki67 in tumour sample (magnification: ×400).

The anti-tumour effect of BSM-0004 has been evaluated *in vitro* using tumorsphere formation assay at 10 µM and 20 µM concentration under normoxic as well as hypoxic conditions. Result evidently suggested that BSM-0004 at 20 µM significantly halted tumorsphere formation of MCF7 cells as compared to untreated cells in both conditions normoxia and hypoxia (Figure 6(A,B)). This experimental evidence clearly indicates the tumoricidal effect of BSM-0004 under hypoxic as well as normoxic conditions. Further, the *in vivo* anti-breast cancer activity of BSM-0004 has been examined at the dose of 15 mg/kg body weight in the breast cancer xenograft model using BALB/c-nu nude mouse. Upon development of tumour model, 15 mg/kg dose of BSM-0004 has been given for 26 days and control animal only received vehicle. The result directed that BSM-0004 significantly reduced tumour volume time-dependently as compared to the control group (*p<.05) (Figure 6(E)). Additionally, BSM-004 treatment significantly (**p<.0012) decreased tumour weight as compared to untreated animals, indicating effective tumoricidal efficacy. It was noted that treatment of BSM-004 decreased almost 50% tumour weight as compared to untreated group’s animals. Remarkably, it was also observed that BSM-0004 treatment for 26 days did not decline the body weight of the animals as compared to vehicle treated group that signifies a non-toxic nature of BSM-0004 (Figure 5(D)). Moreover, BSM-0004 treated animals were examined carefully during the whole experimental period to visualise any significant symptoms associated with toxicity of drug. Notably, BSM-0004 treated animals did not demonstrate any kind of abnormal symptoms associated with toxicity such as alteration in behaviour, low food as well as water intake.

### 3.7. Histopathological examination of xenograft tumour

Furthermore, the *ex vivo* antitumor effect of BSM-0004 was also examined by performing an IHC study of breast cancer biomarker Ki67 and hCA IX. As results shown in Figure 6(H), a significant reduction in expression of Ki67 was observed in the tumour of BSM-0004 treated mouse as compared to the untreated mouse that further indicates the tumoricidal effect of this novel hCA IX inhibitor. A reduced expression of hCA IX has been also seen in the tumour of BSM-0004 treated animals compared to untreated animals indicating down regulation of hCA IX in xenograft mice model. Thus, these results endorse *in vivo* anticancer efficacy of BSM-0004 through hCA IX inhibition.

## 4. Discussion

Indeed existing treatment strategy for cancer is suffering from several complications such as severe side effects, ineffectiveness as well as drug resistance^55^^,^^56^. Therefore, there is an urgent need to explore new targets for the therapy of cancers that may pave the way to overcome drug resistance. In the past decade, hCA IX has been thoroughly studied and validated as an effective druggable target for the therapy of cancers^57^^,^^58^. To impede cancer growth by targeting hCA IX, several inhibitors of this enzyme have been developed in the past years. However, most of them are being studied in the pre-clinical as well as the clinical trial stages^19^. Therefore, the development of new hCA IX inhibitors with promising anticancer activity is still desired.

In this line, we have evaluated *in vitro* as well as *in vivo* anticancer action of novel hCA IX inhibitor BSM-0004. Anticancer effectiveness along with its mechanism of action has been explicated *in vitro* as well as *in vivo*. BSM 0004 has been explored as an effective and selective hCA IX inhibitor with nanomolar range inhibition against hCA IX.

The anti-breast cancer activity of this novel inhibitor has been evaluated against MCF 7 cells as well as MDA-MA-231 cells under normoxic and hypoxic conditions. Several studies indicated overexpression of hCA IX in hypoxic tumours and its inhibition encumbers tumour growth and metastasis. Our result evoked that treatment of BSM-0004 significantly exerted cytotoxic effect against MCF 7 cells under normoxic as well as hypoxic conditions, concentration and time-dependently. IC_50_ values of 35.03 and 30.65 µM were observed for BSM-0004 against MCF 7 cells under normoxic and hypoxic conditions, respectively. Cytotoxic effect on MCF 7 cells was also noticed in phase-contrast microscopy. Additionally, BSM-0004 successfully prohibited colony formation of MCF7 cells, indicating effective antiproliferative action. Further, we premeditated to examine the effect of BSM-004 on the expression of hCA IX in MCF 7 cells under normoxic as well as hypoxic conditions. Interestingly, BSM-0004 significantly reduced expression of hypoxia-induced hCA IX in MCF cells at 72 h.

Uncontrolled cell division promotes the growth and proliferation of cancerous cells. Targeting cell cycle checkpoints is considered an effective tool to control the proliferation of cancer cells and several anticancer agents target various checkpoints of the cell cycle to avert cell division in cancer cells^59^. Thus, the effect of BSM-0004 on cell cycle regulation was analysed under normoxic as well as hypoxic conditions. Noticeably, BSM-004 significantly enhanced MCF 7 cell population in the G2/M phase as compared to untreated cells and thus, successfully arrested the G2/M phase of the cell cycle.

Induction of apoptosis in cancerous cells is an imperious strategy to stop their uncontrolled proliferation and many anticancer drugs follow this pathway to kill cancer cells^60^. Captivatingly, BSM-004 (10 µM and 20 µM) actively induces apoptosis in MCF 7 under normoxic as well as hypoxic conditions as analysed by FACS analysis. Further, DAPI staining also confirmed apoptotic action of BSM-004 in MCF 7 cells in which significant morphological alterations such as chromatin condensation and nuclear fragmentation were visualised upon treatment of this compound in both conditions normoxia as well as hypoxia.

After this, we inspected the effect of BSM-004 in the regulation of anti-apoptotic and pro-apoptotic biomarker proteins that play an important role in cell fate. Anti-apoptotic protein Bcl2 opposes cell death by obstructing the movement of cytochrome c from mitochondria, though pro-apoptotic protein Bax induces apoptosis^61^. It was noticed that BSM-0004 significantly reduces the expression of Bcl2 and augments the expression of Bax in MCF 7 cells under hypoxia conditions.

X-linked inhibitor of apoptosis protein XIAP is known as an inhibitor of apoptosis and its downregulation encourages cell death^62^. Our western blot analysis revealed that BSM-0004 treatment to MCF 7 cell significantly diminishes expression of apoptotic inhibitor XIAP under hypoxia condition. Furthermore, we also investigated the regulation of STAT-3 after treatment of BSM-0004 to MCF 7 cells under hypoxia condition. Evidence directs that STAT-3 significantly contributes to tumourigenesis by up-regulating the expression of several anti-apoptotic proteins. Noticeably, it was found that treatment of BSM-0004 significantly lowered the expression of STAT-3 that further authenticated its anti-proliferative action.

We extended our study to investigate *in vivo* anticancer effect of BSM-0004 against MCF 7-xenograft nude mice model. Palpably, BSM-0004 at the dose of 15 mg/kg/bwt displayed effective anticancer activity against this model and significantly reduced tumour weight and tumour volume as compared to untreated animals. Interestingly, no deaths were recorded in BSM-0004 treated animals during the whole experimental period. Additionally, All BSM-0004 treated animals were found normal and no sign of severe toxicity associated with drug treatment was observed.

Finally, an IHC study of animal’s tumour has been performed to examine the *ex vivo* anticancer effect of BSM-0004. Indeed IHC experiment indicated that BSM-0004 significantly reduced expression of breast cancer biomarker Ki67 as compared to the untreated animals. Markedly, a low expression of hCA IX was also observed in the tumours of BSM-0004 treated animals as compared to the tumours of the untreated group.

Thus, this finding further verifies that the anticancer effect of BSM-0004 is governed by hCA IX inhibitory action. Overall, these *in vivo* experiments visibly indicate that BSM-0004 has promising *in vivo* anticancer activity without any severe side effects.

## 5. Conclusions

Herein, we disclose *in vitro* and *in vivo* anti-breast cancer activity of novel hCA IX inhibitor BSM-0004. BSM-0004 showed effective cytotoxic effect against MCF 7 cells under normoxic as well as hypoxic conditions, being able to impede colony formation of MCF7 cells. This novel hCA IX inhibitor effectively targeted G2/M phase of cell cycle and induced apoptosis in MCF 7 cells under normoxic as well as hypoxic conditions. In addition, this compound promisingly regulated expression of apoptosis associated biomarkers that further authenticates its *in vitro* anticancer effectiveness. Moreover, BSM-0004 halted formation of tumour spheroids of MCF 7 cells under normoxic as well as hypoxic conditions in concentration dependent manner. Noticeably, BSM-0004 significantly inhibited tumour progression in MCF-7 xenograft nude mice model, lowering tumour weight, and tumour volume in BSM-0004 treated mice as compared to untreated mice. Interestingly, reduced expression of cancer biomarkers Ki 67 and hCA IX was noticed in the tumours of BSM-0004 treated animals as compared to untreated animals that further advocate anti-cancer supremacy of this novel compound through hCA IX inhibition. Hence, this investigation offered a promising anti-breast cancer agent which showed effective anti-cancer action *in vitro* and *in vivo*, targeting hCA IX.

## References

[1] Momenimovahe Z, Salehiniya H. Epidemiological characteristics of and risk factors for breast cancer in the world. Breast Cancer 2019;11:151–64. 31040712 10.2147/BCTT.S176070 PMC6462164

[2] Hussain A, Yong C, Tkaczuk KHR, et al. Prevalence and risk of skeletal complications and use of radiation therapy in elderly women diagnosed with metastatic breast cancer. PLOS One 2018;13:e0193661. 29494653 10.1371/journal.pone.0193661 PMC5832309

[3] Fahad UM. Breast cancer: current perspectives on the disease status. Adv Exp Med Biol 2019;1152:51–64. 31456179 10.1007/978-3-030-20301-6_4

[4] Harbeck N, Penault-Llorca F, Cortes J, et al. Breast cancer. Nat Rev Dis Primers 2019;5:66. 31548545 10.1038/s41572-019-0111-2

[5] Sun YS, Zhao Z, Yang ZN, et al. Risk factors and preventions of breast cancer. Int J Biol Sci 2017;13:1387–97. 29209143 10.7150/ijbs.21635 PMC5715522

[6] Veronesi U, Boyle P, Goldhirsch A, et al. Breast cancer. Lancet 2005;365:1727–41. 15894099 10.1016/S0140-6736(05)66546-4

[7] Moo TA, Sanford R, Dang C, Morrow M. Overview of breast cancer therapy. PET Clin 2018;13:339–54. 30100074 10.1016/j.cpet.2018.02.006 PMC6092031

[8] Peart O. Breast intervention and breast cancer treatment options. Radiol Technol 2015;86:535M–58M. 25995413

[9] Redig AJ, McAllister SS. Breast cancer as a systemic disease: a view of metastasis. J Intern Med 2013;274:113–26. 23844915 10.1111/joim.12084 PMC3711134

[10] Borniger JC. Central regulation of breast cancer growth and metastasis. J Cancer Metastasis Treat 2019;5:23. 31773065 10.20517/2394-4722.2018.107 PMC6879058

[11] Mishra CB, Kumari S, Angeli A, et al. Discovery of benzenesulfonamide derivatives as carbonic anhydrase inhibitors with effective anticonvulsant action: design, synthesis, and pharmacological evaluation. J Med Chem 2018;61:3151–65. 29566486 10.1021/acs.jmedchem.8b00208

[12] Mishra CB, Kumari S, Angeli A, et al. Discovery of benzenesulfonamides with potent human carbonic anhydrase inhibitory and effective anticonvulsant action: design, synthesis, and pharmacological assessment. J Med Chem 2017;60:2456–69. 28253618 10.1021/acs.jmedchem.6b01804

[13] Mishra CB, Kumari S, Angeli A, et al. Discovery of potent anti-convulsant carbonic anhydrase inhibitors: design, synthesis, in vitro and in vivo appraisal. Eur J Med Chem 2018;156:430–43. 30015076 10.1016/j.ejmech.2018.07.019

[14] Mishra CB, Kumari S, Angeli A, et al. Design, synthesis and biological evaluation of N-(5-methyl-isoxazol-3-yl/1,3,4-thiadiazol-2-yl)-4-(3-substitutedphenylureido) benzenesulfonamides as human carbonic anhydrase isoenzymes I, II, VII and XII inhibitors. J Enzyme Inhib Med Chem 2016;31:174–9. 27314170 10.1080/14756366.2016.1197221

[15] Rotondi G, Guglielmi P, Carradori S, et al. Design, synthesis and biological activity of selective hCAs inhibitors based on 2-(benzylsulfinyl)benzoic acid scaffold. J Enzyme Inhib Med Chem 2019;34:1400–13. 31401897 10.1080/14756366.2019.1651315 PMC6713143

[16] Uslu AG, Gür Maz T, Nocentini A, et al. Benzimidazole derivatives as potent and isoform selective tumor-associated carbonic anhydrase IX/XII inhibitors. Bioorg Chem 2020;95:103544. 31915112 10.1016/j.bioorg.2019.103544

[17] Scozzafava A, Mastrolorenzo A, Supuran CT. Modulation of carbonic anhydrase activity and its applications in therapy. Exp Opin Ther Pat 2004;14:667–702.

[18] Supuran CT, Scozzafava A. Carbonic anhydrase inhibitors and their therapeutic potential. Exp Opin Ther Patents 2000;10:575–600.

[19] Mishra CB, Tiwari M, Supuran CT. Progress in the development of human carbonic anhydrase inhibitors and their pharmacological applications: where are we today? Med Res Rev 2020;40:2485–565. 32691504 10.1002/med.21713

[20] Guler OO, De Simone G, Supuran CT. Drug design studies of the novel antitumor targets carbonic anhydrase IX and XII. Curr Med Chem 2010;17:1516–26. 20166929 10.2174/092986710790979999

[21] Supuran CT. Carbonic anhydrase inhibition and the management of hypoxic tumors. Metabolites 2017;7:E48. 28926956 10.3390/metabo7030048 PMC5618333

[22] Monti SM, Supuran CT, De Simone G. Carbonic anhydrase IX as a target for designing novel anticancer drugs. Curr Med Chem 2012;19:821–30. 22214452 10.2174/092986712799034851

[23] D’Ascenzio M, Secci D, Carradori S, et al. 1,3-Dipolar cycloaddition, HPLC enantioseparation, and docking studies of saccharin/isoxazole and saccharin/isoxazoline derivatives as selective carbonic anhydrase IX and XII inhibitors. J Med Chem 2020;63:2470–88. 31972093 10.1021/acs.jmedchem.9b01434

[24] Margheri F, Ceruso M, Carta F, et al. Overexpression of the transmembrane carbonic anhydrase isoforms IX and XII in the inflamed synovium. J Enzyme Inhib Med Chem 2016;31:60–3. 27539792 10.1080/14756366.2016.1217857

[25] Supuran CT, Alterio V, Di Fiore A, et al. Inhibition of carbonic anhydrase IX targets primary tumors, metastases, and cancer stem cells: three for the price of one. Med Res Rev 2018;38:1799–836. 29635752 10.1002/med.21497

[26] Singh S, Lomelino CL, Mboge MY, et al. Cancer drug development of carbonic anhydrase inhibitors beyond the active site. Molecules 2018;23:1045. 10.3390/molecules23051045 PMC6099549 29710858

[27] Tafreshi NK, Lloyd MC, Bui MM, et al. Carbonic anhydrase IX as an imaging and therapeutic target for tumors and metastases. Subcell Biochem 2014;75:221–54. 24146382 10.1007/978-94-007-7359-2_12 PMC4282494

[28] Supuran CT. Experimental carbonic anhydrase inhibitors for the treatment of hypoxic tumors. J Exp Pharmacol 2020;12:603–17. 33364855 10.2147/JEP.S265620 PMC7751321

[29] Angeli A, Carta F, Nocentini A, et al. Carbonic anhydrase inhibitors targeting metabolism and tumor microenvironment. Metabolites 2020;10:412. 10.3390/metabo10100412 PMC7602163 33066524

[30] Pastorekova S, Gillies RJ. The role of carbonic anhydrase IX in cancer development: links to hypoxia, acidosis, and beyond. Cancer Metastasis Rev 2019;38:65–77. 31076951 10.1007/s10555-019-09799-0 PMC6647366

[31] Pastorek J, Pastorekova S. Hypoxia-induced carbonic anhydrase IX as a target for cancer therapy: from biology to clinical use. Semin Cancer Biol 2015;31:52–64. 25117006 10.1016/j.semcancer.2014.08.002

[32] Zatovicova M, Jelenska L, Hulikova A, et al. Carbonic anhydrase IX as an anticancer therapy target: preclinical evaluation of internalizing monoclonal antibody directed to catalytic domain. Curr Pharm Des 2010;16:3255–63. 20819068 10.2174/138161210793429832

[33] Winum JY, Scozzafava A, Montero JL, Supuran CT. Inhibition of carbonic anhydrase IX: a new strategy against cancer. Anticancer Agents Med Chem 2009;9:693–702. 19601749 10.2174/187152009788680028

[34] De Simone G, Supuran CT. Carbonic anhydrase IX: biochemical and crystallographic characterization of a novel antitumor target. Biochim Biophys Acta 2010;1804:404–9. 19679200 10.1016/j.bbapap.2009.07.027

[35] Winum JY, Rami M, Scozzafava A, et al. Carbonic anhydrase IX: a new druggable target for the design of antitumor agents. Med Res Rev 2008;28:445–63. 17880011 10.1002/med.20112

[36] Supuran CT, Winum JY. Carbonic anhydrase IX inhibitors in cancer therapy: an update. Future Med Chem 2015;7:1407–14. 26230880 10.4155/fmc.15.71

[37] Thiry A, Dogné JM, Masereel B, Supuran CT. Targeting tumor-associated carbonic anhydrase IX in cancer therapy. Trends Pharmacol Sci 2006;27:566–73. 16996620 10.1016/j.tips.2006.09.002

[38] Supuran CT. Carbonic anhydrase inhibitors as emerging agents for the treatment and imaging of hypoxic tumors. Expert Opin Investig Drugs 2018;27:963–70. 10.1080/13543784.2018.1548608 30426805

[39] Monti SM, Supuran CT, De Simone G. Anticancer carbonic anhydrase inhibitors: a patent review (2008–2013). Expert Opin Ther Pat 2013;23:737–49. 23672415 10.1517/13543776.2013.798648

[40] Poulsen SA. Carbonic anhydrase inhibition as a cancer therapy: a review of patent literature, 2007–2009. Expert Opin Ther Pat 2010;20:795–806. 20476848 10.1517/13543776.2010.484803

[41] Supuran CT, Winum JY. Designing carbonic anhydrase inhibitors for the treatment of breast cancer. Expert Opin Drug Discov 2015;6:591–7. 10.1517/17460441.2015.1038235 25891195

[42] Lock FE, McDonald PC, Lou Y, et al. Targeting carbonic anhydrase IX depletes breast cancer stem cells within the hypoxic niche. Oncogene 2013;32:5210–9. 23208505 10.1038/onc.2012.550

[43] Güttler A, Theuerkorn K, Riemann A, et al. Cellular and radiobiological effects of carbonic anhydrase IX in human breast cancer cells. Oncol Rep 2019;41:2585–94. 30720123 10.3892/or.2019.7001

[44] Ward C, Meehan J, Gray M, et al. Carbonic anhydrase IX (CAIX), cancer, and radiation responsiveness. Metabolites 2018;8:13. 10.3390/metabo8010013 PMC5874614 29439394

[45] Robertson N, Potter C, Harris AL. Role of carbonic anhydrase IX in human tumor cell growth, survival, and invasion. Cancer Res 2004;64:6160–5. 15342400 10.1158/0008-5472.CAN-03-2224

[46] Eldehna WM, Nocentini A, Elsayed ZM, et al. Benzofuran-based carboxylic acids as carbonic anhydrase inhibitors and antiproliferative agents against breast cancer. ACS Med Chem Lett 2020;11:1022–7. 32435420 10.1021/acsmedchemlett.0c00094 PMC7236537

[47] Nocentini A, Supuran CT. Advances in the structural annotation of human carbonic anhydrases and impact on future drug discovery. Expert Opin Drug Discov 2019;14:1175–97. 31436118 10.1080/17460441.2019.1651289

[48] Shaldam M, Eldehna WM, Nocentini A, et al. Development of novel benzofuran-based SLC-0111 analogs as selective cancer-associated carbonic anhydrase isoform IX inhibitors. Eur J Med Chem 2021;216:113283. 33667848 10.1016/j.ejmech.2021.113283

[49] Peppicelli S, Andreucci E, Ruzzolini J, et al. The carbonic anhydrase IX inhibitor SLC-0111 as emerging agent against the mesenchymal stem cell-derived pro-survival effects on melanoma cells. J Enzyme Inhib Med Chem 2020;35:1185–93. 32396749 10.1080/14756366.2020.1764549 PMC7269050

[50] van Kuijk SJA, Parvathaneni NK, Niemans R, et al. New approach of delivering cytotoxic drugs towards CAIX expressing cells: a concept of dual-target drugs. Eur J Med Chem 2017;127:691–702. 27823879 10.1016/j.ejmech.2016.10.037

[51] Mishra CB, Mongre RK, Kumari S, et al. Novel triazole-piperazine hybrid molecules induce apoptosis via activation of the mitochondrial pathway and exhibit antitumor efficacy in osteosarcoma xenograft nude mice model. ACS Chem Biol 2017;12:753–68. 28084722 10.1021/acschembio.6b01007

[52] Mongre RK, Mishra CB, Prakash A, et al. Novel carbazole-piperazine hybrid small molecule induces apoptosis by targeting BCL-2 and inhibits tumor progression in lung adenocarcinoma in vitro and xenograft mice model. Cancers (Basel) 2019;11:1245. 10.3390/cancers11091245 PMC6770606 31450709

[53] Mongre RK, Mishra CB, Jung S, et al. Exploring the role of TRIP-Brs in human breast cancer: an investigation of expression, clinicopathological significance, and prognosis. Mol Ther Oncol 2020;19:105–26. 10.1016/j.omto.2020.09.003 PMC7554327 33102693

[54] Mishra CB, Mongre RK, Kumari S, et al. Synthesis, in vitro and in vivo anticancer activity of novel 1-(4-imino-1-substituted-1H-pyrazolo[3,4-d]pyrimidin-5(4H)-yl)urea derivatives. RSC Adv 2016;6:24491–500.

[55] Maeda H, Khatami M. Analyses of repeated failures in cancer therapy for solid tumors: poor tumor-selective drug delivery, low therapeutic efficacy and unsustainable costs. Clin Transl Med 2018;7:11. 29541939 10.1186/s40169-018-0185-6 PMC5852245

[56] Zhang Z, Zhou L, Xie N, et al. Overcoming cancer therapeutic bottleneck by drug repurposing. Signal Transduct Target Ther 2020;5:113. 32616710 10.1038/s41392-020-00213-8 PMC7331117

[57] Logozzi M, Capasso C, Di Raimo R, et al. Prostate cancer cells and exosomes in acidic condition show increased carbonic anhydrase IX expression and activity. J Enzyme Inhib Med Chem 2019;34:272–8. 30734594 10.1080/14756366.2018.1538980 PMC6327996

[58] Fiaschi T, Giannoni E, Taddei ML, et al. Carbonic anhydrase IX from cancer-associated fibroblasts drives epithelial–mesenchymal transition in prostate carcinoma cells. Cell Cycle 2013;12:1791–801. 23656776 10.4161/cc.24902 PMC3713137

[59] Otto T, Sicinski P. Cell cycle proteins as promising targets in cancer therapy. Nat Rev Cancer 2017;17:93–115. 28127048 10.1038/nrc.2016.138 PMC5345933

[60] Pfeffer CM, Singh ATK. Apoptosis: a target for anticancer therapy. Int J Mol Sci 2018;19:448. 10.3390/ijms19020448 PMC5855670 29393886

[61] Shamas-Din A, Kale J, Leber B, Andrews DW. Mechanisms of action of Bcl-2 family proteins. Cold Spring Harb Perspect Biol 2013;5:a008714. 23545417 10.1101/cshperspect.a008714 PMC3683897

[62] Obexer P, Ausserlechner MJ. X-linked inhibitor of apoptosis protein – a critical death resistance regulator and therapeutic target for personalized cancer therapy. Front Oncol 2014;4:197. 25120954 10.3389/fonc.2014.00197 PMC4112792

